# Human Bocavirus in French Children

**DOI:** 10.3201/eid1208.060213

**Published:** 2006-08

**Authors:** Vincent Foulongne, Yann Olejnik, Virginie Perez, Stéphane Elaerts, Michel Rodière, Michel Segondy

**Affiliations:** * Montpellier University Hospital, Montpellier, France

**Keywords:** human bocavirus, respiratory tract disease, children

## Abstract

Human bocavirus (HBoV), a new member of the genus *Bocavirus* in the family *Parvoviridae*, has been recently associated with respiratory tract infections. We report the epidemiologic and clinical features observed from a 1-year retrospective study of HBoV infection in young children hospitalized with a respiratory tract infection.

Viral respiratory tract infections cause a substantial amount of illness and death in children. Respiratory syncytial virus (RSV), influenza A and B viruses, parainfluenza viruses, human adenoviruses, rhinoviruses, coronaviruses, and the more recently identified human metapneumovirus (HMPV) all cause respiratory tract infections. However, in a substantial proportion of respiratory tract infections, no etiologic agent is detected (*1*), which suggests unknown pathogens. A previously unknown virus likely involved in children's respiratory tract infections has been recently described in Sweden (*2*) and was also identified in Australia (*3*) and Japan (*4*). This newly identified virus shares a high sequence identity and a similar genomic organization with bovine parvovirus and canine minute virus, 2 related members of the *Bocavirus* genus in the *Parvovirinae* subfamily of the *Parvoviridae* family, and it was provisionally named human bocavirus (HBoV).

To investigate epidemiologic features of this virus and further specify clinical signs associated with HBoV infections, we retrospectively tested respiratory specimens from children obtained during a 1-year period. We report the incidence and seasonal distribution of HBoV, provide a phylogenetic analysis of HBoV isolates, and describe clinical characteristics of HBoV infection.

## The Study

The study sample comprised 589 children <5 years of age who were admitted to a pediatric unit of the University Hospital of Montpellier (France) for acute respiratory tract disease, from November 2003 to October 2004. They were 306 boys and 283 girls with a median age of 7 months (range 2 days–60 months). Nasopharyngeal aspirates from these children were tested for common viral respiratory pathogens as previously described (*5*). Samples were tested for respiratory viruses by direct immunofluorescence assays with monoclonal antibodies to RSV; influenza A and B viruses; parainfluenza type 1, 2, and 3 viruses; and human adenovirus. Samples were also injected into MRC5 cell monolayers for virus isolation, and they were tested for HMPV by reverse transcription PCR. Aliquots of samples and of nucleic acid extracts were stored at -80°C.

Nucleic acid extracts were tested for HBoV DNA by PCR with primers targeting the predicted *NP1* gene (*2*). A negative control was included in each PCR run. All HBoV-positive samples were quantitated by real-time PCR with a second sample aliquot. For quantitating HBoV DNA in the positive samples, the 354-bp NP1 PCR fragment was cloned into pGEM-T Easy Vector (Promega, Charbonnières, France). The obtained HBoV-NP1 plasmid was used as control in a subsequent 5´-exonuclease-based real-time PCR assay performed on a LightCycler 2.0 (Roche Diagnostics, Meylan, France) with 2 inner primers (BocaRT1, 5´-CGAAGATGAGCTCAGGGAAT-3´ and BocaRT2, 5´-GCTGATTGGGTGTTCCTGAT-3´) and a FAM/TAMRA dually labeled probe (5´-FAM-CACAGGAGCAGGAGCCGCAG TAMRA-3´). Amplification was performed on 10 μL nucleic acid extract with 0.5 μmol/L both primers and probe and 3 mmol/L MgCl_2_ with FastStart DNA Hybridization Mix (Roche Diagnostics). For quantitation, standard curves were generated by 10-fold dilutions of the pGEM-T HBoV NP1 plasmid. Sensitivity of the PCR assay was 50 copies per reaction as determined by dilutions of the plasmid. DNA level in the HboV-positive samples was measured by using the LightCycler control DNA kit (Roche Diagnostics). Results were expressed as log HBoV DNA copies per nanogram of extracted DNA.

We identified 268 viruses in 259 (44.0%) of the 589 children. Of the 589 children, 165 (28.0%) were infected with RSV, 50 (8.5%) with HMPV, 18 (3.1%) with influenza A viruses, 18 (3.1%) with rhinoviruses, 9 (1.5%) with parainfluenza type 3 viruses, and 8 (1.3%) with human adenoviruses.

**Figure 1:**
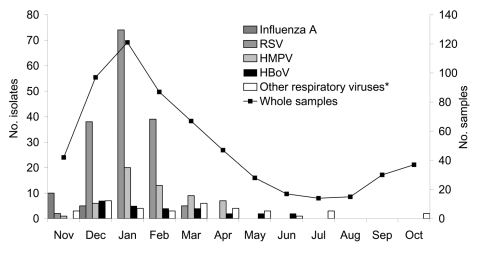
Seasonal distribution of infections caused by human bocavirus (HBoV), human metapneumovirus (HMPV), respiratory syncytial virus (RSV), influenza A virus, and other respiratory viruses during the 1-year study period (November 2003–October 2004). *Other viruses include parainfluenza virus type 3, human adenoviruses, and picornaviruses.

Nasopharyngeal aspirates were positive for HBoV in 26 children (4.4%). Among these HBoV-infected children, 9 (34.6%) were coinfected with another respiratory virus: 5 with RSV, 2 with HMPV, and 2 with human adenovirus. The median HBoV viral load was 2.61 log copies/ng of DNA (range 1.26–4.26 log copies/ng of DNA). The seasonal distribution of respiratory viruses is shown in Figure 1; HBoV was detected from December to June.

**Figure 2:**
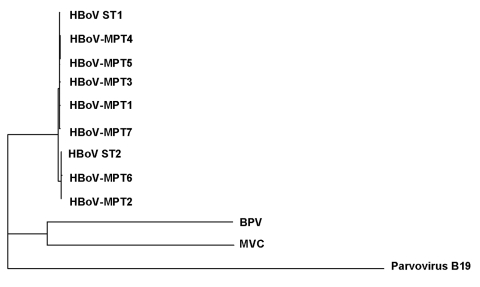
Phylogenetic analysis of human bocavirus (HBoV) *VP2* sequences. Viruses detected in the present study are prefixed HBoV-MPT, followed by the isolate number (GenBank accession nos. AM160609 to AM160615). The nucleotide sequences were aligned with the ClustalW software. Phylogenetic trees were constructed by neighbor-joining through the Institut Pasteur website (http://www.pasteur.fr) with the DNADist and neighbor-joining software packages of the PHYLIP program. The default transition to transversion ratio of 2.0 was retained. We computed 100 bootstrap datasets with random sequence addition to generate consensus trees. BPV, bovine parvovirus; MVC, canine minute virus (minute virus of canine).

Seven HBoV isolates (corresponding to 1 isolate per month) were selected for sequence analysis of a 1-kb DNA fragment encompassing the *VP2* gene. Amplification and sequencing primers were as follows: BocaSEQ1 (5´-AAAATGAACTAGCAGATCTTGATG-3´), BocaSEQ4 (5´-GAACTTGTAAGCAGAAGCAAAA-3´), BocaSEQ2 (5´-GTCTGGTTTCCTTTGTATAGGAGT-3´), and BocaSEQ3 (5´-GACCCAACTCCTATACAAAGGAAAC-3´). These HBoV VP2 sequences were deposited in GenBank under accession numbers AM160609 to AM160615. The nucleotide sequences were aligned, and a phylogenetic tree was constructed to include the 2 previously deposited HBoV sequences ST1 and ST2 as well as the VP2 sequences of canine minute virus (NC_004442) and bovine parvovirus (NC_001540). The VP2 gene of human parvovirus B19 (NC_000883) was chosen as outgroup to root this tree (Figure 2). Our VP2 sequences shared 97.5%–100% nucleotide identity with the HBoV prototype strains ST1 and ST2, whereas amino acid identity was 98.9%–100%.

The 26 HboV-infected children had a median age of 13 months, and the ratio of boys to girls was 1.9. The median duration of hospital stay was 4 days (range 2–39 days). Bronchiolitis was the leading diagnosis, and upper respiratory pathologic features were uncommon. The clinical signs and symptoms among children with HBoV are shown in the Table. The predominant symptoms were dyspnea, respiratory distress, and cough. Half of the children had fever (temperature >38°C). Chest radiographs were obtained for 18 HBoV-infected children, and 15 of them (83.3%) showed abnormal findings, such as hyperinflation or interstitial infiltrates. History of asthma or previous bronchiolitis episodes were reported in 6 HBoV-infected children (23.0%). Three children (11.5%) had an underlying disease (1 congenital heart disease and 2 chronic respiratory diseases), and 7 (26.9%) were born preterm (<36 weeks). Laboratory parameters such as oxygen saturation, C-reactive protein level, or leukocyte count were not relevant in this context.

**Table:** Characteristics of 26 children infected with HBoV*

Characteristic	Value
Demographic data	
Age (mo), median (range)	13 (4–43)
No. boys/no. girls	17/9
Virologic data	
HBoV viral load (log copies/ng DNA), median (range)	2.61 (1.26–4.26)
Viral coinfection,† no. (%)	9 (34.6)
Laboratory findings	
CRP (mg/L), median (range), n = 22	13.5 (<5–166)
Leukocytes (×10^3^/μL), median (range), n = 24	13.2 (7.7–32.0)
SaO_2_ (%), median (range), n = 17	93 (68-99)
Chest radiographic findings, no. (%), n = 18	
Hyperinflation	14 (72.2)
Infiltrate	7 (38.8)
Atelectasis	2 (11.1)
Normal	3 (16.6)
Clinical findings, no. (%)	
Temperature >38°C	13 (50.0)
Cough	13 (50.0)
Dyspnea, wheezing	14 (53.8)
Respiratory distress	14 (53.8)
Rhinorrhea, pharyngitis	8 (30.7)
Otitis	4 (15.4)
Final diagnosis, no. (%)	
Bronchiolitis	12 (46.1)
Pneumonia	3 (11.5)
Asthma	7 (26.9)
Upper respiratory tract infection	4 (15.4)

*HBoV, human bocavirus; CRP, C-reactive protein; SaO_2_, arterial oxygen saturation. †Respiratory syncytial virus (n = 5), human metapneumovirus (n = 2), and adenovirus (n = 2).

## Conclusions

Our data indicate that among children <5 years of age hospitalized with a community-acquired respiratory tract infection, HBoV was detected in 4.4% and represented the third most likely etiologic agent after RSV and HMPV. This 4.4% incidence is in accordance with the values of 3.1%, 5.6%, and 5.7% reported by Allander et al. (*2*), Sloots et al. (*3*), and Ma et al. (*4*). HBoV was detected from December to June, which suggests an epidemiologic difference with respiratory viruses such as RSV, HMPV, or influenza A virus (Figure 1). The absence of HBoV circulation during summer and early fall needs further confirmation. Indeed, this finding might be in part explained by the low number of samples collected during this period.

We previously showed that the HBoV *NP1* gene displayed limited sequence variation (*6*). In the present study, we also observed only minor sequence variations in the *VP2* gene, which suggests that the diverse strains of HBoV belong to a unique lineage. This low genetic diversity of HboV was also reported by Sloots et al., who analyzed the *NS1* gene (*3*).

HBoV infections were mainly identified in children with lower respiratory tract diseases such as bronchiolitis. HBoV was also found in children with asthma exacerbation and, less frequently, in children with upper respiratory infection, which suggests that HBoV shares clinical features with respiratory viruses such as RSV or HMPV (*5**,**7*). Underlying diseases, asthma, or previous bronchiolitis episodes as well as history of prematurity were observed in 61.5% of HBoV-infected children. Even though this feature may be explained by more systematic hospitalization of these children, HBoV pathogenicity may be facilitated by predisposing conditions.

In 9 (34.6%) instances, HBoV was detected concurrently with another respiratory virus. In the original description of HBoV, 17.6% of coinfections involved human adenovirus or RSV (*2*). More recently, a high rate of coinfection (55.6%) was seen in a population of children <3 years of age (*3*). Considering these high rates of coinfection, the exact role played by HBoV in respiratory tract diseases needs to be more precisely defined.
